# Biochanin A Alleviates Cerebral Ischemia/Reperfusion Injury by Suppressing Endoplasmic Reticulum Stress-Induced Apoptosis and p38MAPK Signaling Pathway *In Vivo* and *In Vitro*

**DOI:** 10.3389/fendo.2021.646720

**Published:** 2021-07-12

**Authors:** Min-min Guo, Sheng-biao Qu, Hui-ling Lu, Wen-bo Wang, Mu-Liang He, Jian-Lin Su, Jian Chen, Yong Wang

**Affiliations:** ^1^ Key Laboratory of Tumor Immunology and Microenvironmental Regulation, Guilin Medical University, Guilin, China; ^2^ Department of Pathology, The Second Affiliated Hospital of Kunming Medical University, Kunming, China; ^3^ Department of Pathology and Physiopathology, Guilin Medical University, Guilin, China; ^4^ Department of Neurosurgery, Affiliated Hospital of Guilin Medical University, Guilin, China; ^5^ Department of Neurosurgery, Hezhou Hospital of Traditional Chinese Medicine, Hezhou, China; ^6^ Department of Anesthesiology, Guilin People’s Hospital, Guilin, China; ^7^ Department of Physiology, Guilin Medical University, Guilin, China

**Keywords:** Biochanin A, endoplasmic reticulum stress, apoptosis, p38MAPK, cerebral ischemia/reperfusion, oxygen-glucose deprivation/reoxygenation

## Abstract

We have previously shown that biochanin A exhibits neuroprotective properties in the context of cerebral ischemia/reperfusion (I/R) injury. The mechanistic basis for such properties, however, remains poorly understood. This study was therefore designed to explore the manner whereby biochanin A controls endoplasmic reticulum (ER) stress, apoptosis, and inflammation within fetal rat primary cortical neurons in response to oxygen-glucose deprivation/reoxygenation (OGD/R) injury, and in a rat model of middle cerebral artery occlusion and reperfusion (MCAO/R) injury. For the OGD/R *in vitro* model system, cells were evaluated after a 2 h OGD following a 24 h reoxygenation period, whereas *in vivo* neurological deficits were evaluated following 2 h of ischemia and 24 h of reperfusion. The expression of proteins associated with apoptosis, ER stress (ERS), and p38 MAPK phosphorylation was evaluated in these samples. Rats treated with biochanin A exhibited reduced neurological deficits relative to control rats following MCAO/R injury. Additionally, GRP78 and CHOP levels rose following I/R modeling both *in vitro* and *in vivo*, whereas biochanin A treatment was associated with reductions in CHOP levels but further increases in GRP78 levels. In addition, OGD/R or MCAO/R were associated with markedly enhanced p38 MAPK phosphorylation that was alleviated by biochanin A treatment. Similarly, OGD/R or MCAO/R injury resulted in increases in caspase-3, caspase-12, and Bax levels as well as decreases in Bcl-2 levels, whereas biochanin A treatment was sufficient to reverse these phenotypes. Together, these findings thus demonstrate that biochanin A can alleviate cerebral I/R-induced damage at least in part *via* suppressing apoptosis, ER stress, and p38 MAPK signaling, thereby serving as a potent neuroprotective agent.

## Introduction

Ischemic and hemorrhagic strokes remain a leading cause of death and disability globally. Rapid restoration of normal blood supply following its obstruction or vascular rupture is essential to minimize brain damage and consequent cerebral ischemia/reperfusion (I/R) injury. Cerebral I/R injuries and physiologically complex processes are associated with multiple distinct mechanisms, including oxidative stress, intracellular calcium overload, inflammation, and apoptotic cell death (1, 2).

Recent evidence suggests that endoplasmic reticulum (ER) stress is a key determinant of cerebral I/R injury progression (3, 4). The ER is an organelle that serves as a site of protein folding and as a regulator of calcium homeostasis. Stressful conditions such as ATP depletion, calcium overload, or hypoxia can disrupt normal ER homeostasis and result in misfolded or unfolded protein accumulation. Such ER stress can, when mild, activate the unfolded protein response (UPR) in an effort to restore homeostasis and promote cell survival. The UPR is governed by three regulatory proteins expressed within the ER membrane: activating transcription factor 6 (ATF6), protein kinase RNA-like endoplasmic reticulum kinase (PERK), and inositol-requiring enzyme 1 (IRE1) (5). At baseline, these three proteins bind to the ER chaperone glucose regulated protein 78 (GRP78, also known as binding immunoglobulin protein, BIP) and thereafter remain in an inactive state. In response to protein misfolding, however, GRP78 dissociates from these sensors, leading to their activation and to downstream signaling events that support altered patterns of gene expression that can either drive apoptotic cell death or help mitigate the drivers of ER stress (6, 7). C/EBP-homologous protein; also known as GADD153 (CHOP) is a transcription factor that is activated downstream of these three ER sensor proteins in response to ER stress, whereupon it can inhibit anti-apoptotic Bcl-2 expression, promote caspase activation, and induce apoptotic cell death (8). CHOP and GRP78 levels can thus be used as markers when monitoring ER stress status and cell survival (9). Caspase-12 also serves as an important regulator of ER stress-induced apoptosis, as activated caspase-12 release into the cytoplasm induces the pro-apoptotic activation of caspase-3/9, in turn resulting in DNA fragmentation and apoptotic cell death (10).

Inflammation and ER stress have been linked to one another in a number of studies (11, 12). Notably, inflammation is also a key determinant of cerebral I/R injury outcomes. p38 mitogen-activated protein kinase (p38 MAPK) is a stress-activated kinase that controls cellular differentiation, growth, and response to inflammation (13). The activation of p38 MAPK signaling during cerebral I/R injury can drive the activation of multiple downstream transcription factors that can aggravate brain damage (14, 15). The association between ER stress and MAPK signaling in the context of cerebral I/R injury, however, remains to be fully elucidated.

**Figure 1:**
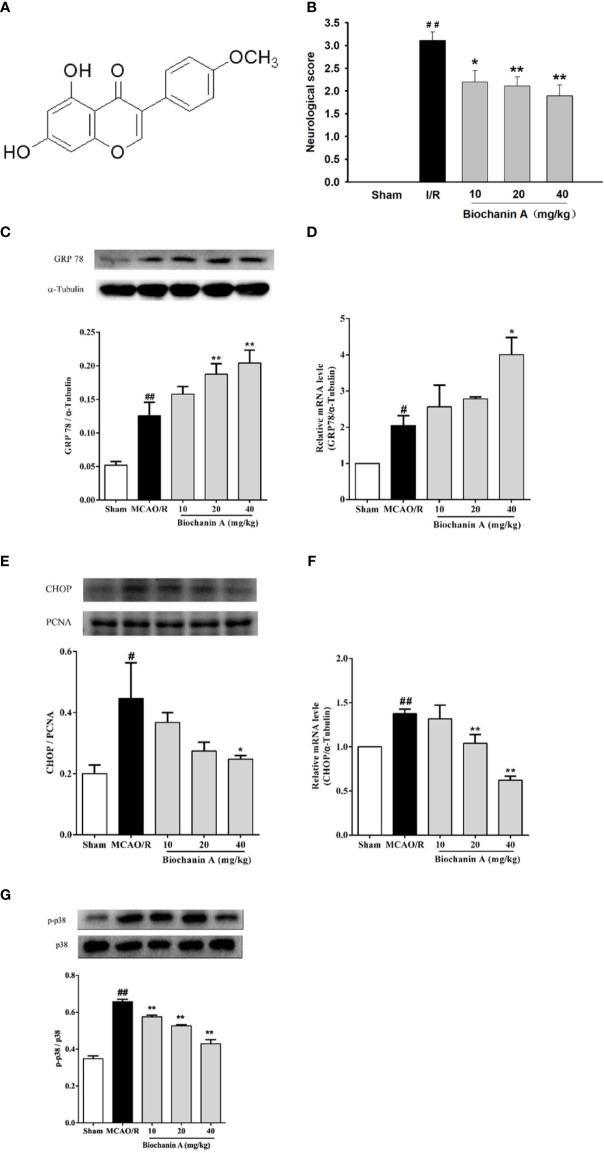
The effects of biochanin A on neuroprotection, attenuating endoplasmic reticulum stress and down-regulating the phosphorylation of p38 expression in MCAO/R rats. **(A)** The basic structure of biochanin A. **(B)** Neurological functional deficits in rats after MCAO/R were assessed using a 5-point scoring system, n=9-10. **(C)** Representative western blot figures and quantification of GRP78 in the rats at 24 h after MCAO/R, n=3. **(D)** GRP78 mRNA expression level in MCAO/R rats, n=3. **(E)** Representative western blot figures and quantification of CHOP in the nuclear fractions of brain tissue at 24 h after MCAO/R, n=3. **(F)** CHOP mRNA expression level in MCAO/R rats, n=3. **(G)** Effects of biochanin A on the activity of p38 in cerebral I/R rats, n=3. The ratio of p-p38 to total p38 was used to evaluate protein activity. Results were presented as mean ± SE. ^#^ *P* < 0.05, ^##^ *P* < 0.01, the MCAO/R group *vs.* the sham group; *P < 0.05, **P < 0.01, *vs.* the MCAO/R group.

Biochanin A (**Figure 1A**) is a primary bioactive isoflavone phytoestrogen that can be isolated from chickpeas and red clover. In prior studies, biochanin A has been shown to exert antioxidant, anti-inflammatory, anti-apoptotic, and antitumor activities (16). We have also previously shown that biochanin A is neuroprotective in a rat cerebral I/R injury model through mechanisms that may be linked with the attenuation of p38 MAPK phosphorylation and associated cerebral inflammation (17). The goal of the present study was to expand upon this prior research in an effort to determine the degree to which ER stress influences cerebral I/R injury and the relationship between such influence and the neuroprotective activities of biochanin A in this pathological context.

## Materials and Methods

### Animals and Reagents

Biochanin A (C_16_H_12_O_5_, molecular weight = 284.26, purity ≥ 98.0%, Stanford Chemicals, USA, **Figure 1A**) was dissolved in dimethyl sulfoxide (DMSO) and stored at 4°C prior to experimental utilization. For this study, Sprague-Dawley (SD) rats (males: 225–285g; females: 200–280g, certificate no. 130217) were obtained from the Experimental Animal Center of Guilin Medical University. The guidelines pertaining to the care and use of animals provided by Guilin Medical University were followed when conducting all studies described herein. All rats were housed in a climate-controlled laboratory (20-25°C, 40-60% humidity, 12 h light/dark cycle) with free food and water access.

### Middle Cerebral Artery Occlusion (MCAO) Modeling and Treatment

In vivo experiment, total 60 male SD rats (aged 6-8 weeks) were used and randomized into five experimental groups: a sham-operated group; a saline-treated I/R group; and biochanin A-treated I/R groups intraperitoneally injected with 10, 20, or 40 mg/kg biochanin A each day for 14 days.

MCAO/R modeling was accomplished by anesthetizing all rats with 1.5% pentobarbital (30 mg/kg) at 1h post-final biochanin or saline dosing. A midline incision was then made in the neck, after which the right common carotid artery (CCA) was exposed, and MCAO was performed *via* inserting a silicon-rubber tip-coated nylon monofilament (Jialing Biotechnology Co. LTD, Guangzhou, China) into the internal carotid artery *via* the CCA until the middle cerebral artery (MCA) was blocked at the origin. Following a 2 h occlusion period, this monofilament was carefully removed to initiate reperfusion (18, 19). Following a 24 h reperfusion period MCAO/R model rats were used for downstream analyses. During surgery, a laser Doppler instrument (Periflux 5000, Sweden) was utilized to monitor ipsilateral regional cerebral blood flow changes in all animals using a probe that was positioned above the skull proximal to the MCA (2 mm posterior and 4 mm lateral to the bregma). Successful modeling was considered to have been achieved when cerebral blood flow was reduced by at least 70%, and was rapidly restored following monofilament removal. In this experiment, 12 rats that did not meet the criteria of modeling or died during surgery were excluded, and 48 rats were finally included in the statistics, with 9-10 rats in each group.

### Assessment of Neurological Deficits and Collection of Brain Tissue Samples

Neurological dysfunction in MCAO/R model rats was evaluated using Longa neurological deficiency scores following a 24 h reperfusion period as follows: 0 points – normal behavior, with no evidence of neurological deficits; 1 point – rats are unable to extend the left forepaw fully, consistent with mild neurological deficits; 2 points – rats circle to the left, consistent with moderate neurological deficits; 3 points – rats fall to the left, consistent with severe neurological deficits; 4 points – rats are unable to walk spontaneously, and/or lose consciousness (18).

After the neurological deficit scoring was completed, the rats were decapitated and the fresh brains were removed quickly. Then brain tissue samples were collected from the ischemic penumbra cortex and striatum of the ipsilateral hemisphere for the subsequent quantitative real-time PCR and western blotting tests.

### Primary Cortical Neuronal Culture and Oxygen-Glucose Deprivation/Reoxygenation (OGD/R) Modeling

*In vitro* experiment, 12 pregnant SD rats on gestational day 17-19 were provided, and a total of 50 rat fetuses were used. Primary cortical neurons were prepared from the SD rat embryo brains. Briefly, the pregnant SD rats were anesthetized and the embryos were detached from the uterus. After the meninges were carefully discarded from the embryo brain, the cortical tissues were harvested and removed into a culture dish filled with DMEM-F12 medium (Boster, China) containing 10% fetal bovine serum. Then the cortical tissues were cut into 0.5-1 mm^3^ small pieces, and digested with trypsin (3-4 ml) (Solarbio, China) and DNA ligase (1-2 ml) (MACKLIN, China) in CO_2_ incubator at 37°C for 20 minutes. Slightly shake the tissues 4 times every 5 minutes to make it fully contact with the enzyme. Digestion was terminated by adding DMEM-F12 medium containing 10% fetal bovine serum for 10 minutes. Tissues were gently stirred with a pipette to yield a uniform suspension. Cells were then seeded on a tissue culture dish that had been pre-coated using Poly-L-Lysine (PLL) (Sigma, USA). 100 × 10^4^ cells were planted in one well of the 6-well plates, 8 × 10^4^ cells in one well of the 96-well plates, 400 × 10^4^ cells in one well of the 6 cm plate. Following a 4 h period, the medium was exchanged for Neurobasal medium (Gibco, USA) containing 2% B27 (Gibco, USA) and 1% glutamine (Gibco, USA). All reagents used for these experiments were phenol red-free.

To simulate cerebral I/R injury *in vitro*, the OGD/R model was established. Briefly, cells were pretreated for 24 h with appropriate biochanin A concentrations. OGD was then induced by culturing primary cortical neurons in glucose-free EBSS and incubated in a hypoxic chamber (Billups-Rothenberg, CA, USA) in a 5% CO_2_ and 95% N_2_ atmosphere at 37°C for 2 h or other appropriate time. For control cells, glucose-containing EBSS was instead used. After this OGD period, cells were returned to complete Neurobasal medium and incubated at 37°C for 24 h in a standard humidified incubator to mimic reperfusion. Control cells underwent a similar procedure, but did not undergo OGD/R.

### Immunofluorescence Imaging

Neuron purity was established *via* neuron-specific enolase (NSE) immunofluorescent labeling. Primary cortical neurons were cultured for 5 days on coverslips, after which they were rinsed using PBS (3 times for 5 minutes each time), fixed for 30 minutes with 4% paraformaldehyde at 4°C , permeabilized for 10 minutes with 0.2% Triton X-100, blocked for 1 h at room temperature with 5% bovine serum albumin (BSA), and then incubated overnight with anti-NSE (AF2164, Beyotime, Shanghai, China, 1:200) at 4°C. Meanwhile, rabbit’ serum control group was set up in parallel. After the primary antibody was incubated, it was rinsed with PBS 3 times, 5 minutes each time. Coverslips were then probed for 1 h with an appropriate secondary antibody at 37°C while protected from light, after which 4’,6-diamidino-2-phenylindole (DAPI) (C0060, Solarbio, Beijing, China, 1:100) was used to counterstain these coverslips. Cells were then evaluated *via* fluorescence microscopy, with DAPI-positive nuclei (blue fluorescence, represents the total number of cells) and NSE-positive cytoplasmic area (green fluorescence, represents the number of primary cortical neurons) being used to identify neurons. Numbers of DAPI- and NSE-positive cells in the same field of view were determined and compared in order to establish neuronal purity.

### Cell Viability Analyses

A CCK-8 assay kit(CK04, Dojindo, Shanghai, China)was used to measure cell viability based on provided directions. Briefly, primary cortical neurons cultured for 5 days were implanted on 96-well plates (8×10^4^/well), and were then treated with three different doses of biochanin A (2 μM, 4 μM, 8 μM) for 24 h, and subjected to OGD and 24 h reoxygenation as an experimental model. After treatment and OGD/R modeling, CCK-8 solution was added to appropriate wells, and plates were incubated for 4 h at 37°C, after which absorbance was measured at 450 nm with a microplate reader (Bio-Rad Model 680, Bio-Rad, CA, USA). Viability was then determined as follows: [(mean experimental absorbance - mean blank absorbance)/(mean control absorbance - mean blank absorbance)] × 100%. Cell viability was expressed as a percentage compared with 100% in the control group.

### Quantitative Real-Time PCR (qRT-PCR)

Trizol (Invitrogen) was used to extract total RNA from tissue or cell samples, after which a Revert Aid First Strand cDNA Synthesis Kit (TIANGEN, Beijing, China) was used to prepare cDNA. All qRT-PCR reactions were conducted using an ABI PRISM 7500 Sequence Detector System (Applied Biosystems, CA, USA), and relative gene expression was quantified *via* the 2^–ΔΔCt^ approach, with α-Tubulin being used as a normalization control. The primer sequences used in the qRT-PCR were as follows:

GRP78, 5’-CAAGAACCAACTCACGTCCA-3’(forward) and
5’-CCACCTTGAATGGCAAGAAC-3’(reverse);
CHOP, 5’-GTTGGCATCACCTCCTGTCT-3’(forward) and
5’-CCCTCTCCTTTGGTCTACCC-3’(reverse);
α-Tubulin, 5’-TGTCACCAACTGGGACGATA-3’(forward) and
5’-GGGGTGTTGAAGGTCTCAAA-3’(reverse).

### Western Blotting

Cells or brain tissue samples were homogenized in chilled RIPA buffer to extract total protein, whereas nuclear or cytosolic proteins were separately isolated using cytoplasmic or nuclear protein extraction kits (Beyotime Biotechnology, China) as appropriate. A BCA assay (Beyotime Biotechnology) was then used to measure protein levels in extract samples, after which equivalent protein amounts (30 μg/sample) were separated *via* SDS-PAGE and transferred to 0.45 μm PVDF membranes (Bio-Rad Laboratories, USA). These blots were then blocked for 1 h using 5% non-fat milk in TBST (tris-buffered saline containing 0.1% Tween-20) at 37°C, after which they were incubated at 4°C overnight with appropriate primary antibodies: anti-GRP78 (sc-13968, Santa Cruz Biotechnology, Dallas, TX, USA, 1:200), anti-CHOP (15204-1-AP, Proteintch Group, Wuhan, China, 1:200), anti-p38 (9212, Cell Signaling Technology, Danvers, USA, 1:1000), anti-p-p38 (4511, Cell Signaling Technology, Danvers, USA, 1:1000), anti-caspase-3 (19677-1-AP, Proteintch Group, Wuhan, China, 1:500), anti-caspase-12 (ab62484, Abcam, Cambridge, UK, 1:500), anti-Bcl-2 (12789-1-AP, Proteintch Group, Wuhan, China,1:1000), anti-Bax (50599-2-lg, Proteintch Group, Wuhan, China, 1:2000), anti-β-actin (TA-09, ZSGB-Bio, Beijing, China, 1:1000), anti-α-Tubnin (AF7010, Abcam, Cambridge, UK, 1:1000), anti-PCNA (A01040, Abbkine, Wuhan, China, 1:1000). The following day, blots were probed for 1 h with secondary antibodies at 37°C, after which the Super Signal West Pico Western blot detection reagent (Bridgen, China) was used to detect protein bands, which were subsequently imaged with the Image Lab software (Bio-Rad, USA).

### Statistical Analysis

Data are means ± SE and were assessed using SPSS 17.0 (SPSS, IL, USA). The one-way ANOVA (analysis of variance) followed by a post-hoc LSD (least significant difference) test was performed for statistical comparison of individual groups. A difference of *P* < 0.05 was considered to indicate a statistically significant.

## Results

### Biochanin A Attenuates MCAO-Induced Neurological Impairment

In an effort to establish the neuroprotective activity of biochanin A in an animal model of cerebral I/R injury, we administered biochanin A (10, 20, or 40 mg/kg qd) to rats for 14 days prior to MCAO modeling. While sham-operated control rats did not exhibit any neurological impairment, MCAO/R model rats exhibited substantial neurological deficits. Treatment with biochanin A significantly attenuated these deficits in a dose-dependent manner (**Figure 1B**). These data thus indicated that biochanin A exerts a neuroprotective role in this rat model of cerebral I/R.

### Biochanin A Suppresses Endoplasmic Reticulum Stress and p38 MAPK Activation in MCAO/R Model Rats

We next evaluated the impact of biochanin A treatment on endoplasmic reticulum stress by assessing CHOP and GRP78 mRNA and protein levels in MCAO/R model rats. Relative to sham controls, MCAO/R model rats exhibited significant increases in both GRP78 and CHOP expression. Biochanin A treatment further increased GRP78 mRNA and protein expression while suppressing CHOP mRNA and protein levels in a dose-dependent manner (**Figures 1C–F**). This indicates that biochanin A is capable of suppressing ER stress in the context of cerebral I/R injury.

Prior studies have also demonstrated that p38 MAPK activation in the pathogenesis of cerebral I/R. As such, we evaluated p38 phosphorylation in cerebral tissue samples from these experimental animals. This analysis revealed that p38 phosphorylation levels were significantly higher in MCAO/R model rats relative to sham controls, whereas biochanin A treatment was linked to the dose-dependent suppression of this phosphorylation in MCAO/R model animals (**Figure 1G**).

### Biochanin A Suppresses Apoptosis in MCAO/R Rats

**Figure 2:**
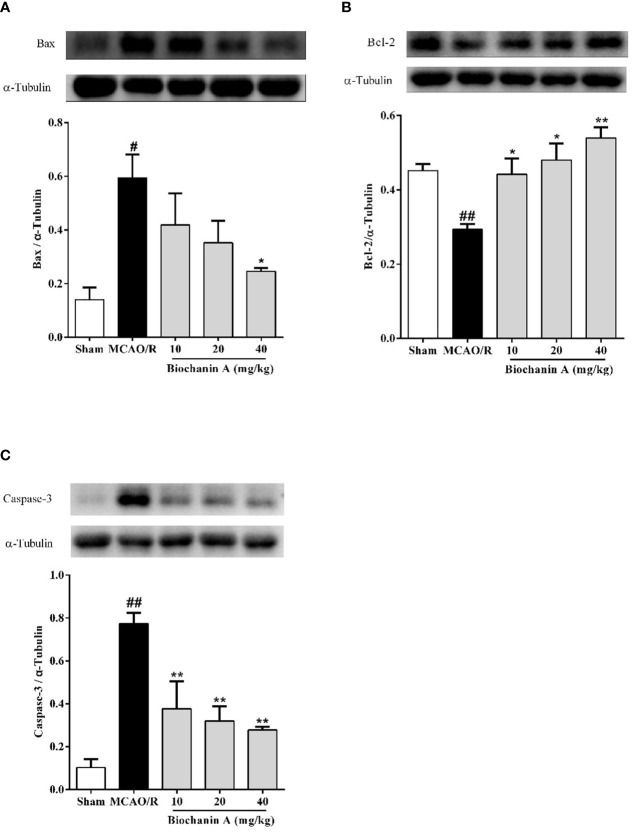
The effects of biochanin A on apoptosis in the cerebral cortical penumbra following MCAO/R in rats. The protein expressions of Bax **(A)**, Bcl-2 **(B)** and Caspase-3 **(C)** were measured by Western blot and quantified by densitometric analyses in cerebral I/R rats. α-Tubulin were used as internal controls. Results were presented as mean ± SE, n = 3. ^#^ *P* < 0.05, ^##^ *P* < 0.01, the MCAO/R group *vs.* the sham group; *P < 0.05, **P < 0.01, *vs.* the MCAO/R group.

To evaluate the impact of biochanin A treatment on apoptosis in the context of cerebral I/R injury we also quantified caspase-3, Bcl-2, and Bax levels in brain tissue samples from these MCAO/R model rats. Relative to sham controls, Bax and caspase-3 levels were significantly increased in MCAO/R model animals, whereas Bcl-2 levels were markedly reduced. Biochanin A treatment, however, reversed these changes in a dose-dependent fashion (**Figures 2A–C**), indicating that this phytoestrogen functions as an inhibitor of apoptosis in cerebral I/R rats.

### Biochanin A Enhances Primary Cortical Neuron Proliferation Following OGD/R

**Figure 3:**
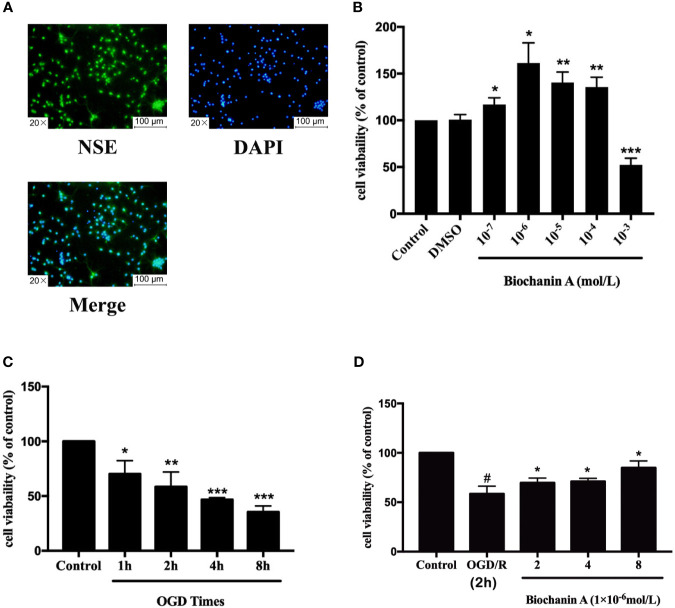
The optimum of concentrations for biochanin A and OGD times on primary cortical neurons. **(A)** Identification of primary neurons. **(B)** Effects of biochanin A at different concentrations on primary neurons. **(C)** Effects of different OGD times on primary neurons. **(D)** Effects of biochanin A at different concentrations on primary neurons after 2 h of OGD. Cell viability was detected by CCK8 assay. Results are expressed as the mean ± SE, n = 3. *P < 0.05, **P < 0.01, ***P < 0.001, *vs.* the control group or OGD/R group. ^#^P < 0.05, the OGD/R group *vs*. the control group.

We next conducted *in vitro* experiments using cortical neurons, which were assessed for purity following a 5-day culture period based on immunofluorescent NSE staining, which revealed these cells to be 93.59 ± 0.01% (n = 12) pure (**Figure 3A**). The viability of these cells was then evaluated *via* CCK8 assay following OGD/R and biochanin A treatment. This analysis revealed that treatment of these neurons with biochanin A concentrations from 10^-7^ - 10^-4^ mol/L was sufficient to enhance neuronal growth, with maximal growth enhancement at a concentration of 10^-6^ mol/L (**Figure 3B**). As biochanin A concentrations rose to 10^-3^ mol/L, this compound induced cytotoxic neuronal death. As such, concentrations of (2~8)×10^-6^ mol/L of biochanin A were used for subsequent experiments. Following a 1~8 h exposure to OGD conditions, primary cortical neuronal injury was also evident *via* CCK8 assay (**Figure 3C**). A 2 h OGD period was selected for subsequent experiments as it was associated with a moderate level of cellular injury (57% viability). Following 2h OGD period, cells were allowed to undergo reperfusion for 24 h. Experimental results revealed that biochanin A was able to improve neuronal survival in response to OGD treatment in a dose-dependent manner (**Figure 3D**). Biochanin A can thus promote primary neuron survival following OGD/R.

### Biochanin A Alleviates OGD/R-Induced ER Stress and p38 Activation *In Vitro*

**Figure 4:**
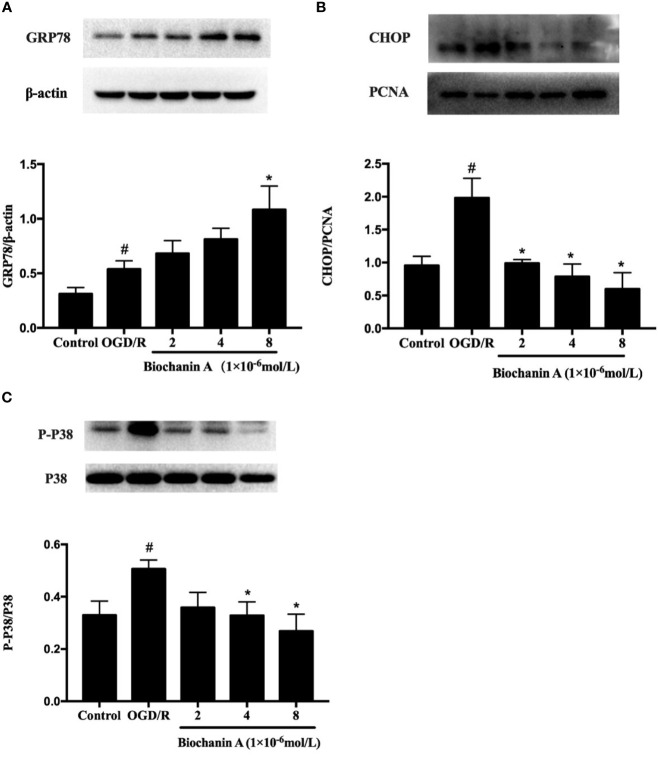
Biochanin A ameliorated endoplasmic reticulum stress and inhibited the phosphorylation activity of p38 in OGD/R neurons. **(A)** Protein expression of GRP78 in primary cortical neurons with OGD/R induction. β-actin were used as internal controls. **(B)** Protein expression of CHOP in primary cortical neurons with OGD/R induction. PCNA were used as internal controls. **(C)** Effects of biochanin A on the phosphorylation activity of p38 in primary cortical neurons after OGD/R. The ratio of p-p38 to total p38 was used to evaluate protein activity. Results were presented as mean ± SE, n = 3. ^#^P < 0.05, the OGD/R group *vs.* the control group; *P < 0.05, *vs.* the OGD/R group.

To expand upon the above results, we additionally evaluated the impact of biochanin A treatment in this *in vitro* OGD/R experimental system. We found that CHOP and GRP78 protein levels rose significantly following OGD/R treatment relative to control treatment, whereas biochanin A treatment increased GRP78 expression and reduced CHOP expression in a dose-dependent fashion (**Figures 4A, B**). We also found that OGD/R was associated with a significant increase in p38 phosphorylation in cultured neurons, while biochanin A treatment reversed this effect in a dose-dependent manner, consistent with *in vivo* results (**Figure 4C**). As such, biochanin A is capable of alleviating OGD/R-induced ER stress and suppressing p38 MAPK-induced inflammation in this experimental context.

### Biochanin A Reduces OGD/R-Induced Apoptosis in Primary Cortical Neurons

**Figure 5:**
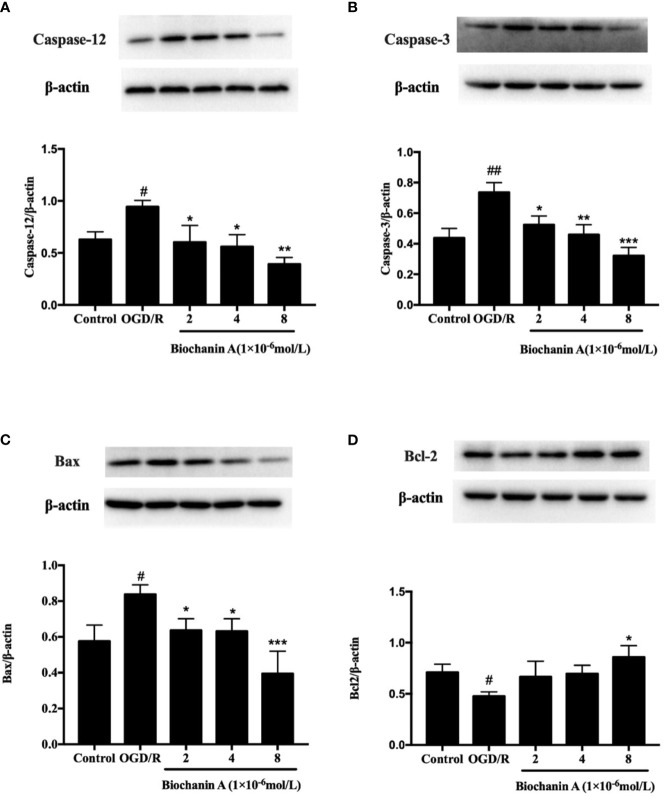
The effects of biochanin A on apoptosis-related proteins in primary cortical neurons with OGD/R induction. Protein expression levels of caspase-12 **(A)**, capase-3 **(B)**, Bax **(C)** and Bcl-2 **(D)** were assessed by Western blot. β-actin were used as internal controls. Results were presented as mean ± SE, n = 3. ^#^ *P* < 0.05, ^##^ *P* < 0.01, the OGD/R group *vs.* control group; *P < 0.05, **P < 0.01, ***p < 0.001, *vs.* the OGD/R group.

Lastly, we assessed the impact of biochanin A on the apoptotic death of primary cortical neurons following OGD/R exposure. We found that OGD/R was associated with significant increases in caspase-3, caspase-12, and Bax levels and with reduced Bcl-2 levels relative to control treatment, whereas biochanin A reversed these effects (**Figures 5A–D**). These data suggest that OGD/R drives apoptotic cell death *via* ER-related caspase-12 activation, while biochanin A is able to suppress this ER stress response and to thereby decrease apoptotic cell death in the context of OGD/R injury.

## Discussion

ER stress responses have increasingly been linked to the pathological basis for ischemic stroke. However, the mechanisms whereby ER stress regulates brain injury in this pathological context remain to be clarified. Phytoestrogens have been shown to exert neuroprotective functions in multiple studies to date, and the potential pharmacological mechanisms of these natural compounds needs further investigate. In the present study, we determined that ER stress occurs in the context of cerebral I/R injury. In addition, the results of this analysis indicated that biochanin A, which is an isoflavone phytoestrogen derived from chickpeas and other leguminous plants, is neuroprotective in the context of I/R injury both *in vitro* and *in vivo* through mechanisms associated with ER stress alleviation, suppression of ER stress-related apoptosis, and inhibition of p38 MAPK signaling.

ER stress can be initiated in response to multiple factors including ischemia, hypoxia, and metabolic dysfunction, and prolonged deleterious ER stress is believed to contribute to the pathology of diabetes, cancer, neurodegenerative disorders, and ischemic diseases. GRP78 is activated in response to ER stress, initiating the UPR following its release from stress receptor proteins. The activation of GRP78 has previously been shown to protect neurons against apoptosis and excitotoxicity, whereas its inhibition can exacerbate neuronal death *via* disrupting calcium homeostasis and enhancing oxidative stress (20). The GRP78 inducer BiP inducer X (BIX) can, consequently, suppress apoptosis in response to ER stress *in vivo* and *in vitro* (21). There is also prior evidence that GRP78 protects neural stem cells against OGD/R-induced cell death through the regulation of PI3K/Akt, ERK1/2, and NF-κB/p65 (22). In the present report, we found that biochanin A enhanced the expression of GRP78 in the context of cerebral I/R injury, thereby helping to restore ER homeostasis and support neuronal survival.

CHOP is a multifunctional transcription factor capable of activating the death-receptor, mitochondrial, and ER stress-related apoptotic pathways. Under physiological conditions, CHOP is expressed at relatively low levels in most cells. It is rapidly and markedly upregulated, however, in response to apoptotic or ER stress signaling following signaling mediated by the ER stress sensor proteins IRE1, ATF6, and PERK. In response to ER stress, these sensors become active, ultimately driving the nuclear localization of activating transcription factor 4 (ATF4), cleaved ATF6α, and x-box binding protein-1, (XBP-1), which induce CHOP expression (23, 24). CHOP, in turn, promotes the upregulation of pro-apoptotic proteins including Bax, Bim, Bid, Bad, Bak, and Puma, as well as the downregulation of anti-apoptotic Bcl-2 and Bcl-xl. CHOP also coordinates intracellular calcium signaling and induces the upregulation of death receptor 4 (DR4) and DR5, thus coordinating the apoptotic response (25). CHOP has been shown to be specifically activated in the ER stress-induced apoptosis following cerebral I/R injury (26), and such ischemia related neuronal death is attenuated in CHOP-deficient mice (27).

IRE1 also functions as an ER stress receptor protein that can recruit TNF receptor-associated factor 2 (TRAF2) and thereby activate apoptotic-signaling kinase-1(ASK1), ultimately resulting in JNK or p38 MAPK signaling (28). CHOP can also undergo p38 MAPK-mediated phosphorylation, further enhancing its stress-induced pro-apoptotic activity (29). As such, this IRE1-ASK1-p38 pathway may be a central driver of CHOP-mediated apoptotic cell death in response to endogenous or exogenous stressors. Indeed, recent work suggests that p38 MAPK activation within pancreatic cancer-derived exosomes can promote ER stress and consequent T cell apoptosis (30). Another study has shown that p38 activates ER stress-ATF6α axis, which plays a crucial role in cellular senescence (31). Therefore, the p38 MAPK signaling pathway is closely related to the activation of ER stress. Whether a similar mechanism may also underlie the role of p38 MAPK and ER stress responses in the context of cerebral I/R injury, however, remains uncertain. Nonetheless, such findings provide a point of reference for future studies of how p38MAPK induces CHOP-mediated apoptosis.

Caspase-12 is an interleukin-1β converting enzyme (ICE) caspase subfamily member that primarily localizes to the ER and that plays important roles in the context of ER stress-induced apoptosis and inflammation (32). Following the induction of ER stress, the release of Ca^2+^ from the ER lumen results in the activation of proteases including caspase-7 and calpain, which in turn cleave pro-caspase-12 into its active form (33). Once activated, caspase-12 subsequently activates caspase-9 and caspase-3 through cytochrome c-independent mechanisms, thereby driving apoptosis (10). Several studies to date have identified changes in the expression of ER stress markers as well as caspase-12 expression and activation in the context of cerebral ischemia in rodent model systems both *in vitro* and *in vivo* (26, 34, 35). Inhibiting ER stress and associated apoptotic cell death can attenuate brain injury in experimental cerebral I/R models (36, 37). In the present analysis, we found that CHOP, Bax, and activated caspase-12 and caspase-3 levels rose significantly following OGD/R or MCAO/R treatment, whereas Bcl-2 expression declined under these conditions. This suggests that I/R-induced injury is associated with ER stress and associated apoptotic cell death. We further found that p38 MAPK activation was markedly increased following cerebral I/R injury, indicating that ERS-induced apoptosis and inflammation are closely associated with such activation. Biochanin A treatment was sufficient to reverse these I/R injury-induced changes in apoptosis-related marker expression and p38 MAPK activation. While the mechanistic basis for the neuroprotective activity of biochanin A remains to be fully elucidated, our data nonetheless provide a theoretical basis for future studies of the means by which this phytoestrogen protects against ER stress-induced apoptosis in the context of ischemic stroke.

However, some limitation should be noted. Firstly, the design of this study is a prophylactic administration of biochanin A, and whether it can play a therapeutic role after cerebral ischemia reperfusion injury remains to be observed and verified. There is still a long way to go towards clinical application. Secondly, biochanin A, as one of the phytoestrogens, needs to be further tested to see whether the above neuroprotective mechanisms against cerebral ischemic reperfusion injury is exerted through estrogen receptor-associated pathways. Thirdly, this study only observed the effects of biochanin A on ER-stress and apoptotic signaling, but failed to explore the underlying mechanism behind these effects.

In conclusion, biochanin A attenuates ER stress-induced apoptosis and p38 MAPK activation in cerebral ischemia reperfusion injury *in vivo* and *in vitro*. The neuroprotective mechanisms of biochanin A may be related to suppressing ER stress, apoptosis and inflammatory response. Our results lay a foundation for further research on the role and mechanism of biochanin A, and provide evidences for the development of phytoestrogens in the prevention and treatment of ischemic stroke.

## Data Availability Statement

The original contributions presented in the study are included in the article/supplementary material. Further inquiries can be directed to the corresponding authors.

## Ethics Statement

The animal study was reviewed and approved by Guilin Medical University Animal Care and Use Committee.

## Author Contributions

JC and YW conceived the project and supervised all experiments. M-mG, S-bQ, H-lL, and W-bW performed the experiments. JC, YW, M-lH, and J-lS acquired and analyzed the data. JC and YW wrote and revised the manuscript. All authors contributed to the article and approved the submitted version.

## Funding

This work was supported by the National Natural Science Foundation of China (nos. 81560378 and 81860231), and the Guangxi Natural Science Foundation (nos. 2018GXNSFAA050054 and 2018GXNSFAA294138), and the Independent Research Project of Guangxi Key Laboratory of Tumor Immunology and Microenvironmental Regulation (no. 203030301806), and the Scientific Research Ability Enhancement Project for Young and Middle-aged Faculty of Guilin Medical University (no. 20502018006).

## Conflict of Interest

The authors declare that the research was conducted in the absence of any commercial or financial relationships that could be construed as a potential conflict of interest.

## References

[1] Kalogeris T Baines CP Krenz M Korthuis RJ. Ischemia/Reperfusion. Compr Physiol (2016) 7(1):113–70. 10.1002/cphy.c160006 PMC5648017 28135002

[2] Campbell BCV De Silva DA Macleod MR Coutts SB Schwamm LH Davis SM. Ischaemic Stroke. Nat Rev Dis Primers (2019) 5(1):70. 10.1038/s41572-019-0118-8 31601801

[3] Xin Q Ji B Cheng B Wang C Liu H Chen X. Endoplasmic Reticulum Stress in Cerebral Ischemia. Neurochem Int (2014) 68:18–27. 10.1016/j.neuint.2014.02.001 24560721

[4] Yang W Paschen W. Unfolded Protein Response in Brain Ischemia: A Timely Update. J Cereb Blood Flow Metab (2016) 36(12):2044–50. 10.1177/0271678X16674488 PMC5363674 27733676

[5] Oakes SA Papa FR. The Role of Endoplasmic Reticulum Stress in Human Pathology. Annu Rev Pathol (2015) 10:173–94. 10.1146/annurev-pathol-012513-104649 PMC5568783 25387057

[6] Ibrahim IM Abdelmalek DH Elfiky AA. GRP78: A Cell’s Response to Stress. Life Sci (2019) 226:156–63. 10.1016/j.lfs.2019.04.022 PMC7094232 30978349

[7] Casas C. GRP78 at the Centre of the Stage in Cancer and Neuroprotection. Front Neurosci (2017) 11:177. 10.3389/fnins.2017.00177 28424579 PMC5380735

[8] McCullough KD Martindale JL Klotz LO Aw TY Holbrook NJ. Gadd153 Sensitizes Cells to Endoplasmic Reticulum Stress by Down-Regulating Bcl2 and Perturbing the Cellular Redox State. Mol Cell Biol (2001) 21(4):1249–59. 10.1128/MCB.21.4.1249-1259.2001 PMC99578 11158311

[9] Schonthal AH. Endoplasmic Reticulum Stress and Autophagy as Targets for Cancer Therapy. Cancer Lett (2009) 275(2):163–9. 10.1016/j.canlet.2008.07.005 18692955

[10] Morishima N Nakanishi K Takenouchi H Shibata T Yasuhiko Y. An Endoplasmic Reticulum Stress-Specific Caspase Cascade in Apoptosis. Cytochrome C-Independent Activation of Caspase-9 by Caspase-12. J Biol Chem (2002) 277(37):34287–94. 10.1074/jbc.M204973200 12097332

[11] Sprenkle NT Sims SG Sanchez CL Meares GP. Endoplasmic Reticulum Stress and Inflammation in the Central Nervous System. Mol Neurodegener (2017) 12(1):42. 10.1186/s13024-017-0183-y 28545479 PMC5445486

[12] Santos LE Ferreira ST. Crosstalk Between Endoplasmic Reticulum Stress and Brain Inflammation in Alzheimer’s Disease. Neuropharmacology (2018) 136(Pt B):350–60. 10.1016/j.neuropharm.2017.11.016 29129774

[13] Coulthard LR White DE Jones DL McDermott MF Burchill SA. P38(MAPK): Stress Responses From Molecular Mechanisms to Therapeutics. Trends Mol Med (2009) 15(8):369–79. 10.1016/j.molmed.2009.06.005 PMC3016890 19665431

[14] Han D Scott EL Dong Y Raz L Wang R Zhang Q. Attenuation of Mitochondrial and Nuclear P38alpha Signaling: A Novel Mechanism of Estrogen Neuroprotection in Cerebral Ischemia. Mol Cell Endocrinol (2015) 400:21–31. 10.1016/j.mce.2014.11.010 25462588

[15] Ashraf MI Ebner M Wallner C Haller M Khalid S Schwelberger H. A P38mapk/MK2 Signaling Pathway Leading to Redox Stress, Cell Death and Ischemia/Reperfusion Injury. Cell Commun Signal (2014) 12:6. 10.1186/1478-811X-12-6 24423080 PMC3896752

[16] Sarfraz A Javeed M Shah MA Hussain G Shafiq N Sarfraz I. Biochanin A: A Novel Bioactive Multifunctional Compound From Nature. Sci Total Environ (2020) 722:137907. 10.1016/j.scitotenv.2020.137907 32208265

[17] Wang W Tang L Li Y Wang Y. Biochanin A Protects Against Focal Cerebral Ischemia/Reperfusion in Rats via Inhibition of P38-Mediated Inflammatory Responses. J Neurol Sci (2015) 348(1-2):121–5. 10.1016/j.jns.2014.11.018 25466482

[18] Longa EZ Weinstein PR Carlson S Cummins R. Reversible Middle Cerebral Artery Occlusion Without Craniectomy in Rats. Stroke (1989) 20(1):84–91. 10.1161/01.str.20.1.84 2643202

[19] Guo M Lu H Qin J Qu S Wang W Guo Y. Biochanin A Provides Neuroprotection Against Cerebral Ischemia/Reperfusion Injury by Nrf2-Mediated Inhibition of Oxidative Stress and Inflammation Signaling Pathway in Rats. Med Sci Monit (2019) 25:8975–83. 10.12659/MSM.918665 PMC6896748 31767824

[20] Yu Z Luo H Fu W Mattson MP. The Endoplasmic Reticulum Stress-Responsive Protein GRP78 Protects Neurons Against Excitotoxicity and Apoptosis: Suppression of Oxidative Stress and Stabilization of Calcium Homeostasis. Exp Neurol (1999) 155(2):302–14. 10.1006/exnr.1998.7002 10072306

[21] Kudo T Kanemoto S Hara H Morimoto N Morihara T Kimura R. A Molecular Chaperone Inducer Protects Neurons From ER Stress. Cell Death Differ (2008) 15(2):364–75. 10.1038/sj.cdd.4402276 18049481

[22] Liu Q Li Y Zhou L Li Y Xu P Liu X. GRP78 Promotes Neural Stem Cell Antiapoptosis and Survival in Response to Oxygen-Glucose Deprivation (OGD)/Reoxygenation Through PI3K/Akt, ERK1/2, and NF-Kappab/P65 Pathways. Oxid Med Cell Longev (2018) 2018:3541807. 10.1155/2018/3541807 29849883 PMC5914129

[23] Oyadomari S Mori M. Roles of CHOP/GADD153 in Endoplasmic Reticulum Stress. Cell Death Differ (2004) 11(4):381–9. 10.1038/sj.cdd.4401373 14685163

[24] Yang Y Liu L Naik I Braunstein Z Zhong J Ren B. Transcription Factor C/EBP Homologous Protein in Health and Diseases. Front Immunol (2017) 8:1612. 10.3389/fimmu.2017.01612 29230213 PMC5712004

[25] Nishitoh H. CHOP is a Multifunctional Transcription Factor in the ER Stress Response. J Biochem (2012) 151(3):217–9. 10.1093/jb/mvr143 22210905

[26] Nakka VP Gusain A Raghubir R. Endoplasmic Reticulum Stress Plays Critical Role in Brain Damage After Cerebral Ischemia/Reperfusion in Rats. Neurotox Res (2010) 17(2):189–202. 10.1007/s12640-009-9110-5 19763736

[27] Tajiri S Oyadomari S Yano S Morioka M Gotoh T Hamada JI. Ischemia-Induced Neuronal Cell Death is Mediated by the Endoplasmic Reticulum Stress Pathway Involving CHOP. Cell Death Differ (2004) 11(4):403–15. 10.1038/sj.cdd.4401365 14752508

[28] Homma K Katagiri K Nishitoh H Ichijo H. Targeting ASK1 in ER Stress-Related Neurodegenerative Diseases. Expert Opin Ther Targets (2009) 13(6):653–64. 10.1517/14728220902980249 19456270

[29] Wang XZ Ron D. Stress-Induced Phosphorylation and Activation of the Transcription Factor CHOP (GADD153) by P38 MAP Kinase. Science (1996) 272(5266):1347–9. 10.1126/science.272.5266.1347 8650547

[30] Shen T Huang Z Shi C Pu X Xu X Wu Z. Pancreatic Cancer-Derived Exosomes Induce Apoptosis of T Lymphocytes Through the P38 MAPK-Mediated Endoplasmic Reticulum Stress. FASEB J (2020) 34(6):8442–58. 10.1096/fj.201902186R 32350913

[31] Kim HS Kim Y Lim MJ Park YG Park SI Sohn J. The P38-Activated ER Stress-ATF6alpha Axis Mediates Cellular Senescence. FASEB J (2019) 33(2):2422–34. 10.1096/fj.201800836R 30260700

[32] Garcia de la Cadena S Massieu L. Caspases and Their Role in Inflammation and Ischemic Neuronal Death. Focus on Caspase-12. Apoptosis (2016) 21(7):763–77. 10.1007/s10495-016-1247-0 27142195

[33] de la Cadena SG Hernandez-Fonseca K Camacho-Arroyo I Massieu L. Glucose Deprivation Induces Reticulum Stress by the PERK Pathway and Caspase-7- and Calpain-Mediated Caspase-12 Activation. Apoptosis (2014) 19(3):414–27. 10.1007/s10495-013-0930-7 24185830

[34] Zhao Y Fang Y Zhao H Li J Duan Y Shi W. Chrysophanol Inhibits Endoplasmic Reticulum Stress in Cerebral Ischemia and Reperfusion Mice. Eur J Pharmacol (2018) 818:1–9. 10.1016/j.ejphar.2017.10.016 29031902

[35] Wang K Lou Y Xu H Zhong X Huang Z. Harpagide From Scrophularia Protects Rat Cortical Neurons From Oxygen-Glucose Deprivation and Reoxygenation-Induced Injury by Decreasing Endoplasmic Reticulum Stress. J Ethnopharmacol (2020) 253:112614. 10.1016/j.jep.2020.112614 32007630

[36] Kong T Qiu K Liu M Cheng B Pan Y Yang C. Orexin-A Protects Against Oxygen-Glucose Deprivation/Reoxygenation-Induced Cell Damage by Inhibiting Endoplasmic Reticulum Stress-Mediated Apoptosis *via* the Gi and PI3K Signaling Pathways. Cell Signal (2019) 62:109348. 10.1016/j.cellsig.2019.109348 31233841

[37] Chen Y Zhang L Gong X Gong H Cheng R Qiu F. Iridoid Glycosides From Radix Scrophulariae Attenuates Focal Cerebral Ischemiareperfusion Injury *via* Inhibiting Endoplasmic Reticulum Stressmediated Neuronal Apoptosis in Rats. Mol Med Rep (2020) 21(1):131–40. 10.3892/mmr.2019.10833 PMC6896402 31746404

